# Exploring the ‘cold/hot’ properties of traditional Chinese medicine by cell temperature measurement

**DOI:** 10.1080/13880209.2020.1732429

**Published:** 2020-03-02

**Authors:** Suyun Yu, Can Li, Yushi Ding, Shuai Huang, Wei Wang, Yuanyuan Wu, Fangxu Wang, Aiyun Wang, Yuexia Han, Zhiguang Sun, Yin Lu, Ning Gu

**Affiliations:** a Jiangsu Key Laboratory for Pharmacology and Safety Evaluation of Chinese Materia Medica, Jiangsu Collaborative Innovation Center of Traditional Chinese Medicine Prevention and Treatment of Tumor, School of Pharmacy, Nanjing University of Chinese Medicine, Nanjing, China; b School of Artificial Intelligence and Information Technology, Nanjing University of Chinese Medicine, Nanjing, China; c The State Key Laboratory of Bioelectronics and Jiangsu Key Laboratory of Biomaterials and Devices, School of Biological Sciences and Medical Engineering of Southeast University, Nanjing, China; d Collaborative Innovation Center of Suzhou Nano Science and Technology, Suzhou, China; e Jiangsu Provincial Second Chinese Medicine Hospital, The Second Affiliated Hospital of Nanjing University of Chinese Medicine, Nanjing, China

**Keywords:** Real-time cell temperature, melanoma, wireless thermometry, calcium ion, TRPV4

## Abstract

**Context:**

It is common sense that chewing a mint leaf can cause a cooling feeling, while chewing ginger root will produce a burning feeling. In Traditional Chinese Medicine (TCM), this phenomenon is referred to as ‘cold/hot’ properties of herbs. Herein, it is reported that TCM with different “cold/hot” properties have different effects on the variation of cells.

**Objective:**

To explore the intrinsic ‘cold/hot’ properties of TCM from the perspective of cellular and molecular biology.

**Materials and methods:**

A375 cells were selected using Cancer Cell Line Encyclopaedia (CCLE) analysis and western blots. Hypaconitine and baicalin were selected by structural similarity analysis from 56 and 140 compounds, respectively. A wireless thermometry system was used to measure cellular temperature change induced by different compounds. Alteration of intracellular calcium influx was investigated by means of calcium imaging.

**Results:**

The IC_50_ values of GSK1016790A, HC067047, hypaconitine, and baicalin for A375 cells are 8.363 nM, 816.4 μM, 286.4 μM and 29.84 μM, respectively. And, 8 μM hypaconitine induced obvious calcium influx while 8 μM baicalin inhibited calcium influx induced by TRPV4 activation. Cellular temperature elevated significantly when treated with GSK1016790A or hypaconitine, while the results were reversed when cells were treated with HC067047 or baicalin.

**Discussion and conclusions:**

The changes in cellular temperature are speculated to be caused by the alteration of intracellular calcium influx mediated by TRPV4. In addition, the ‘cold/hot’ properties of compounds in TCM can be classified by using cellular temperature detection.

## Introduction

Syndrome differentiation is a basic principle in Chinese medicine used to understand and treat diseases. The ‘cold/hot’ property of TCM is one of the important theoretical bases in diagnosis, differentiation, and treatment of diseases. However, there is still no convenient method to objectively distinguish between ‘cold’ and ‘hot’ natural herbs.

Previous studies (Zhao et al. 2011) have manifested that mice treated by ‘cold/hot’ natural herbs exhibit different properties in tropism towards cold or hot environments. It is known that mammals need ‘transient receptor potential cation channels’ (TRPs) to response temperature, pain, permeability, taste, touch and other stimuli (Clapham 2003). Eight of these TRPs are proposed to be involved in thermosensation and can be activated under different temperatures. For instance, TRPV1 is activated by moderate heat (>43 °C), while TRPM8 is activated around 15–28 °C. The TRPV1 (>43 °C), TRPV2 (>52 °C), TRPV3 (>33 °C), TRPV4 (28–42 °C), TRPM2 (35–40 °C), and TRPM5 (25–35 °C) channels have incompletely overlapping functions and broad activation temperature ranges from warm to hot. Relatively, cool and cold temperatures can be sensed by TRPM8 (15–28 °C) and TRPA1 (<18 °C) family members (Clapham 2007; Belvisi et al. 2011; Gees et al. 2012). In addition to being activated by temperature, TRPs can also be activated by some small molecule compounds, allowing calcium-based cations to rapidly enter the cytoplasm to maintain osmotic pressure stability and promote signal transmission (Grace et al. 2013; Nilius and Szallasi 2014). The representative ‘hot’ compound capsaicine is an agonist of TRPV1, while the representative ‘cold’ compound menthol is the agonist of TRPM8 (Wetsel 2011). Many compounds extracted from TCM also present some ‘cold/hot’ properties, some of which could produce a burning or cooling feeling in the body such as capsaicin and menthol (Zhao et al. 2009). Compounds from TCM with this characteristic may regulate thermogenesis through TRPs.

The correlation between TRPs and energy metabolism has been extensively studied over the last few decades (Uchida et al. 2011; Wang and Siemens 2015). Studies have reported that the intracellular concentration of Ca^2+^ is relevant to thermogenesis (Uchida et al. 2018). A series of ion channels are mainly involved in a plethora of Ca^2+^-mediated cell functions, whether the opening or closing of the TRPs could alter cell temperature still awaits to be explored. Many TRPs show constitutive activity in overexpression systems. However, only a few have been studied in their native environment or at physiological temperatures, including TRPV3 and TRPV4 channels (Belvisi et al. 2011). Compared with TRPV3 channel, TRPV4 is more widely distributed in various tissues of the body (Gees et al. 2012). Also, TRPV4 has been reported as a regulator of adipose oxidative metabolism and energy homeostasis (Ye et al. 2012).

It was not until the introduction of thermometers that any exact data on the temperature of animals could be obtained. We have developed a wireless thermometry system which can measure real-time temperature on the thousand-cell level using thermal resistors in our previous studies, and it can be used for cell temperature measurements in an incubator (Li et al. 2019a, 2019b). The wireless thermometry consists of twelve thermal resistors for highly concentrated, small-volume cell suspensions seeded on a sensor surface to force the cells to grow only on the surface; a current direction-switching circuit to reduce ambient noise; a multichannel acquisition system for simultaneous multichannel data acquisition; and a transceiver and signal processing system for transmitting the data out from the incubator. The system is designed to be used in an incubator that ensures the proper growing conditions for cells. Cellular temperature variations could be monitored with an accuracy resolution below 0.01 °C. The changes in cellular temperature and concentration of Ca^2+^ may help understand the inherent ‘cold/hot’ properties of compounds.

Therefore, we screened melanoma cell line A375 which has a high expression level of TRPV4, and studied the cellular temperature change when TRPV4 was activated by GSK1016790A or inhibited by HC067047. Furthermore, hypaconitine and baicalin were filtrated as the representative ‘cold/hot’ compounds for subsequent experiments. The temperature changes and intracellular influx of Ca^2+^ were assessed when A375 cell was treated with hypaconitine and baicalin. Cellular temperature was recorded periodically. This is the first time that the wireless temperature measurement technology is used to observe the changes in cell temperature caused by compounds from TCM. And the ‘cold/hot’ properties of compounds may be classified by the cellular temperature changes induced by the compounds themselves.

## Materials and methods

### CCLE analysis

CCLE provides public access to mRNA expression data of approximately 1457 cancer cell lines. A total of 1020 independent cancer cell lines were profiled at the genomic level (data available at http://www.broadinstitute.org/ccle). Gene expression analysis was conducted using the Gene Chip Human Genome U133 Plus 2.0 Array.

### Western blot

Cells at densities of 4 × 10^5^ cells/well were lysed with RIPA buffer (Beyotime, P0013B, Beijing, China). The protein level was measured with the assistance of a BCA assay kit (Thermo Fisher, NCI3227CH, Waltham, MA), and total proteins of approximately 25 μg were separated by SDS-PAGE and then transferred to polyvinylidene uoride membranes (Millipore, Billerica, MA). After labelling with primary and secondary antibodies (TRPV4, ABCAM, ab39260) (Goat Anti-Rabbit IgG (H + L) HRP, Affinity, S0001), the membranes were scanned using a BIORAD imaging system (chemiDOCTMXRS, Bio-Rad, Hercules, CA).

### Cell culture

The immortalized human keratinocyte cell line HaCaT and human melanoma cell A375 were cultured in Dulbecco’s Modified Eagle’s Medium (DMEM; Gibco, Carlsbad, CA) supplemented with 10% foetal bovine serum (FBS; Cellmax, SA301.02, Sunnyvale, CA). The human melanoma cell Malme-3M was cultured in Iscove’s Modified Dubecco’s Medium (IMDM; Gibco, Carlsbad, CA) with 20% FBS, and Skmel-24 was cultured in Modified Eagle’s Medium (MEM; Gibco, Carlsbad, CA) with 15% FBS. All cells were incubated and maintained in a 37 °C incubator with 5% CO_2_.

### Structural similarity compounds screening

This study is based on the DISCOVERY STUDIO 4.0, with subset of the libraries selected to find similar molecules by means of fingerprints function. A library was constructed by combining multiple ligands as reference. Then, the similarity between target molecules and library was compared, and the scoring method was applied to reverse search for drug targets. Similarity >0.5 indicates that the structure of two compounds is spatially similar, and a higher value of similarity indicates a higher structural similarity between the two compounds. The results were obtained by finding similar molecules through the fingerprint, which was the coefficient based on the fingerprint similarity search.

### Cell viability and proliferative measurement

Twenty-four hours prior to drugs treatment, the cells were seeded in 96-well plates at a density of 10^4^/well with 200 μL culture medium, containing 10% FBS. HC067047, hypaconitine, baicalin and GSK1016790A diluted in 2 μL DMSO (Thermo, 20688, Waltham, MA) were added through culture medium replacement. After treatment for 24 h, 3-(4,5-dimethylthiazol-2-yl)−2,5-diphenyltetrazolium bromide (MTT, 35R0GR001) assays were performed according to the standardized procedures. For the modified MTT assays, the blank wells were not added with any drugs, and the control well was added only with 2 μL DMSO. The absorbance of the sample was measured at wavelength of 490 nm with the assistance of a microplate reader (BIOTEK, 270133,  Winooski, VT). Cell viability was calculated as a percentage of the value compared to the control group.

### Cellular temperature measurement

The cell temperature was measured using the wireless thermometry system (Figure 2(A)). The wireless thermometry system was composed of a six-well plate integrated with 12 thin film platinum resistor sensors, a signal acquisition and transmission system, a signal receiving system and a PC for signal processing. As reported in our previous study, the resistance resolution of the circuit is 20 mΩ, which corresponds to no more than 0.01 °C. The resistance deviations of each channel are corrected with software compensation. The linearity between the temperature and the resistance of the sensors lies above 0.999 in the applied temperature range (30–42 °C).

After the cells were confluent on the sensor surface, the temperature experiments were carried out. Before the temperature measurement, the drug was preheated until it reached room temperature (25 °C). Then, the drug was added to the cell medium at the final required drug concentration when the cell reached a steady state (i.e., small temperature fluctuations). Then, the culture board and measuring circuit were placed in the incubator. The temperatures of the cell suspension (*t*_control_) and cell (*t*_cell with suspension_) were tested simultaneously. The temperature changes in pure cells were calculated in accordance with Δ*t* = *t*_cell with suspension_ – *t*_control_. The experiments were independently performed in triplicate.

### Calcium imaging

Intracellular calcium of A375 cells was measured using a similar method as previously reported by Tonello et al. (2017). Plated cells loaded with 2 μM Fura-2 AM-ester (Beyotime, S1052, Beijing, China) were added to the buffer solution (37 °C) containing the following constituents (in mM: 2 CaCl_2_; 5.4 KCl; 0.4 MgSO_4_; 135 NaCl; 10 d-glucose) and10 HEPES, as well as 0.1% bovine serum albumin at pH 7.4. After 30 min of loading, A375 cells were washed and transferred to the chamber on the stage of a Nikon Eclipse TE-2000 U microscope for recording. The cells were excited alternately at 340 and 380 nm to indicate the relative intracellular calcium changes by the ratio 340/380 recorded with a dynamic image analysis system. Cells were treated with TRPV4 agonist-GSK1016790A and ‘hot compound’-hypaconitine to confirm the effect of compounds on intracellular calcium influx. To dissect the intracellular calcium response caused by TRPV4 inhibitor-HC067047 and ‘cold compound’-baicalin, the two compounds were pre-incubated with cells for 30 min before stimulated by GSK1016790A or hypaconitine. Buffer solution containing DMSO 0.1% was used as vehicle.

### Statistical

All results are presented in the form of mean ± SD. Statistical analysis was conducted using Graphpad 7.0 (GraphPad Software, La Jolla, CA). Group change comparison was performed with one-way ANOVA, followed by Tukey tests. *p* Value less than 0.05 was considered statistically significant.

## Results

### Analysis of the TRPV4 expression and screening of representative compounds

In view of the wide variety of temperature sensitive receptors, taking the activation temperature and receptor distribution into account, we chose TRPV4 as a representative receptor to evaluate the relationship between intracellular calcium ion and cellular thermogenesis.

**Figure 1.**
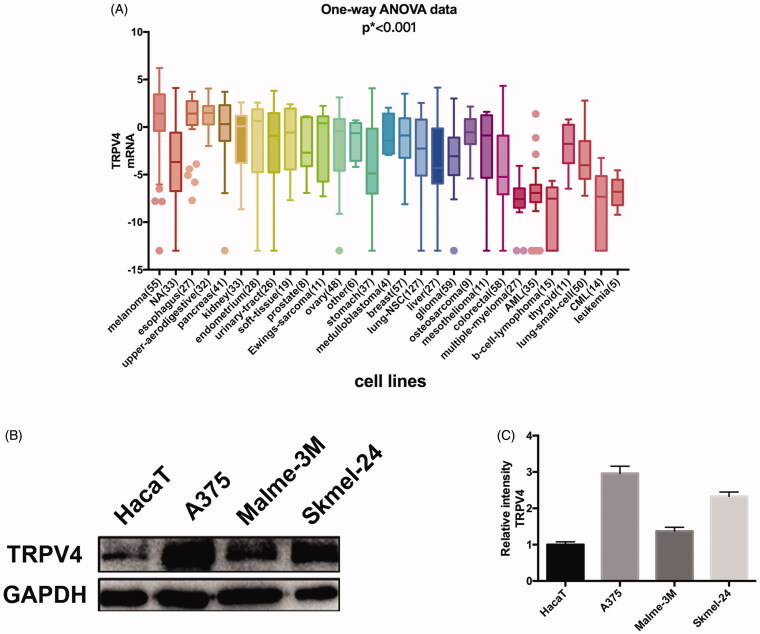
Screening of cells and compounds. (A) mRNA expression of TRPV4 in different types of cells. (B) The protein expression of TRPV4 in melanoma cells compared with epidermal cell, of which A375 was the most significant. (C) Digitalized data from densitometric analysis of the western blots. Values are presented as mean ± SD.

To guarantee the results of subsequent experiments to be more sensitive and accurate, the cells with a high expression level of TRPV4 were selected. The mRNA expression of TRPV4 in cells from various sources were analyzed on the basis of CCLE database and melanoma cells displayed the highest expression for TRPV4 (Figure 1(A)). Then we chose three melanoma cells lines (A375, Malme-3M, and Skmel-24) with high malignancy and one normal epidermal cell (HaCaT) to test and verify the protein expression of TRPV4. Compared with HaCaT cells, the protein level of TRPV4 in melanoma cells was observed to be significantly higher, and A375 exhibited the highest expression for TRPV4 among them (Figure 1(B,C)).

As a temperature-sensitive receptor, whether TRPV4 could be activated/inhibited by some other ‘cold/hot’ compounds in addition to existing agonist/inhibitors remains worthy of exploration. It is common sense that chewing mint leaves could cause a cool feeling, while chewing a piece of ginger root will produce a burning sensation (Zhao et al. 2011). TCM refers to this phenomenon as the hot and cold property of herbal medicine and use it to successfully treat certain diseases (Liang et al. 2013).

The similarity law proposed by Johnson et al. (1989) suggests that the compounds with similar chemical structures possess similar properties. We analyzed the structural similarity of 56 common compounds that represent warm/hot Chinese herbals with GSK1016790A which is the agonist of TRPV4 (Watanabe et al. 2002). As indicated by the results, 11 compounds showed a relatively high structural similarity to GSK1016790A (similarity > 0.5; Table 1). Despite benzoylnapelline demonstrated the highest chemically structural similarity with GSK1016790A, mostly it is metabolized to hypoaconitine which has secondary structural similarity. The structural similarity analysis of the main compounds of 140 representative cool/cold herbal medicines with TRPV4 inhibitor-HC067047, 14 compounds exhibited a relatively high structural similarity to HC067047 (similarity > 0.5; Table 2). The chemical structures of these compounds can also be viewed in Tables 1 and 2. Among them, the structural similarity of baicalin to HC067047 reached 0.62. The similarity means that hot components have similar chemical structures to GSK1016790A, while cold components have similar chemical structures to HC067047. Based on the above experimental results, we selected A375 cell as the experimental cell line, while hypaconitine and baicalin were taken as candidate compounds.

**Table 1.** Structural similarity between GSK1016790A and ‘hot’ compounds from TCM (similarity > 0.5).

Molecule ID	Molecule name	Similarity	Structure
N/A	GSK1016790A	1.00	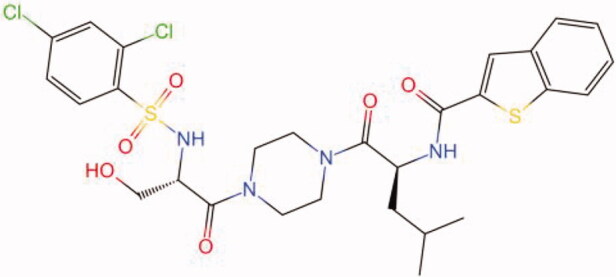
MOL002410	Benzoylnapelline	0.57	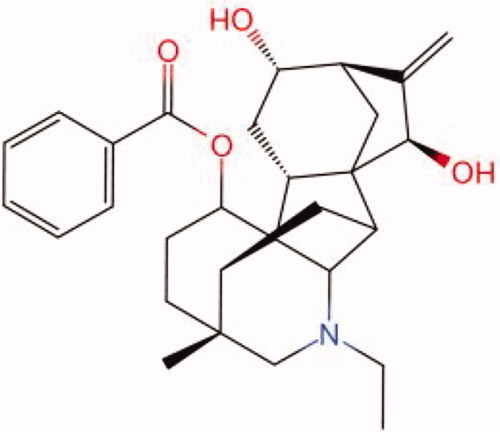
MOL000538	Hypaconitine	0.56	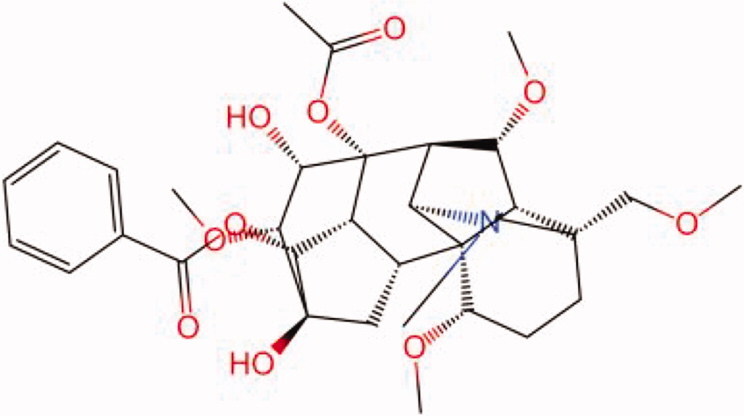
MOL002708	Precarthamin	0.56	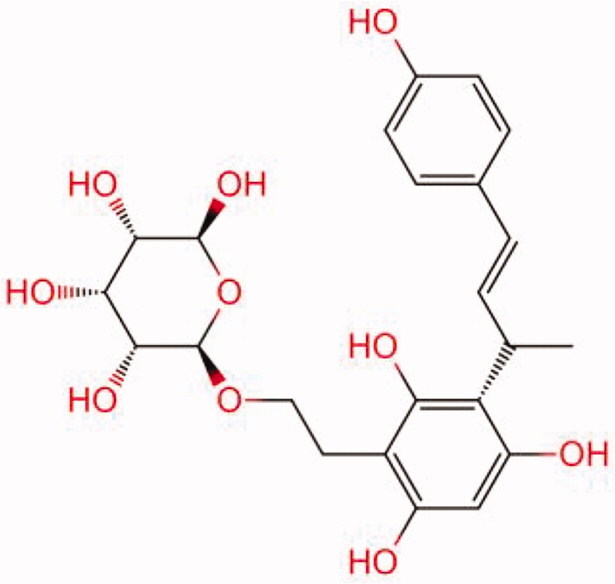
MOL011974	Notopterol	0.56	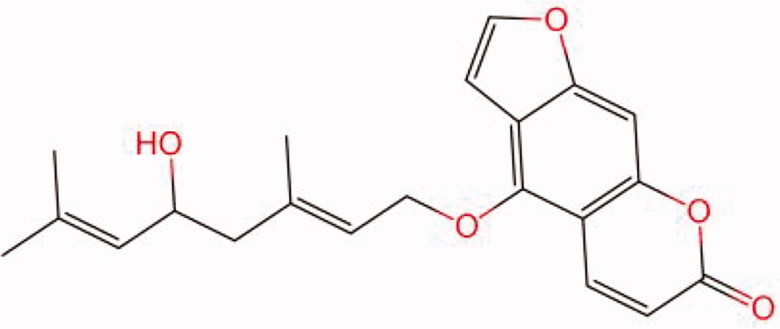
MOL011975	Notoptol	0.55	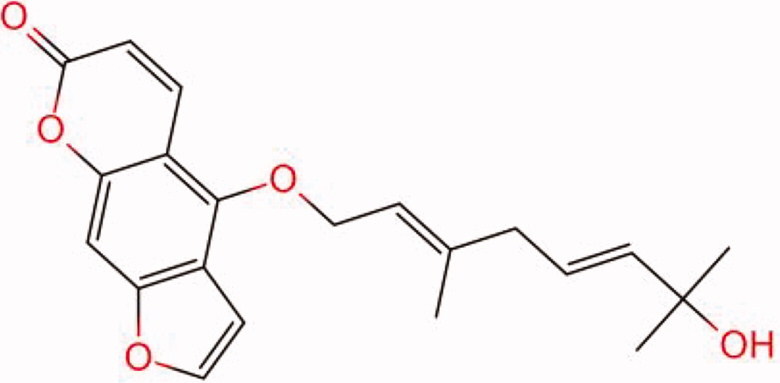
MOL002088	Aconitine	0.55	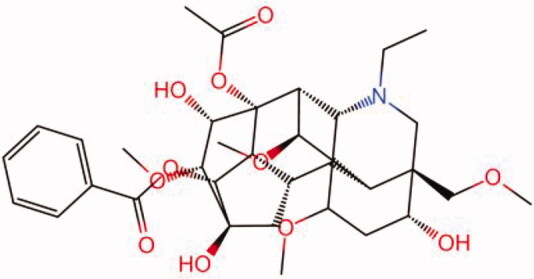
MOL002581	Curcumin	0.55	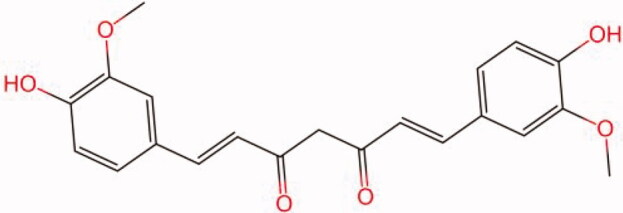
MOL003958	Evodiamine	0.55	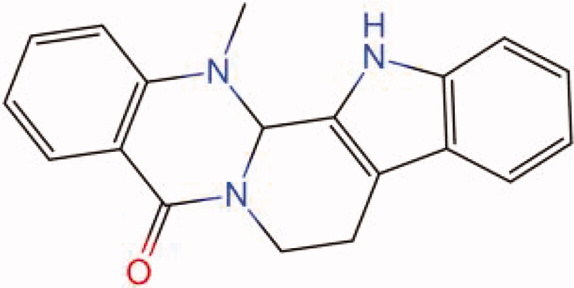
MOL002411	Chasmaconitine	0.54	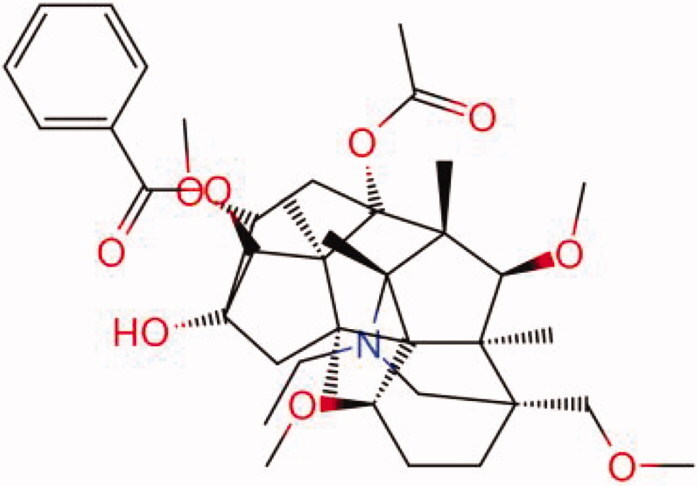
MOL003436	Isobrucine	0.54	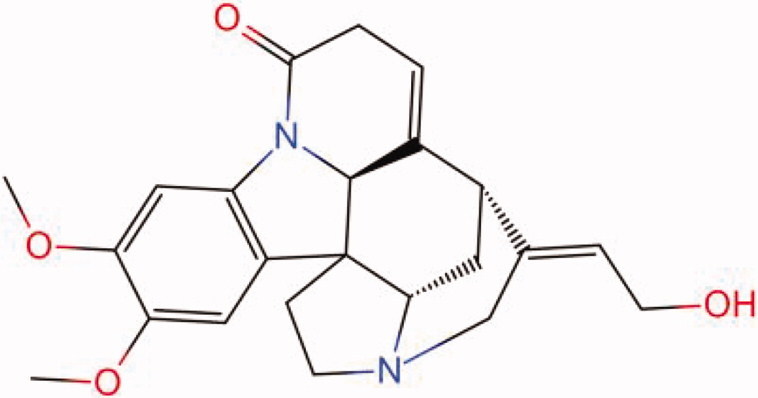
MOL005584	Yuanhuacin	0.52	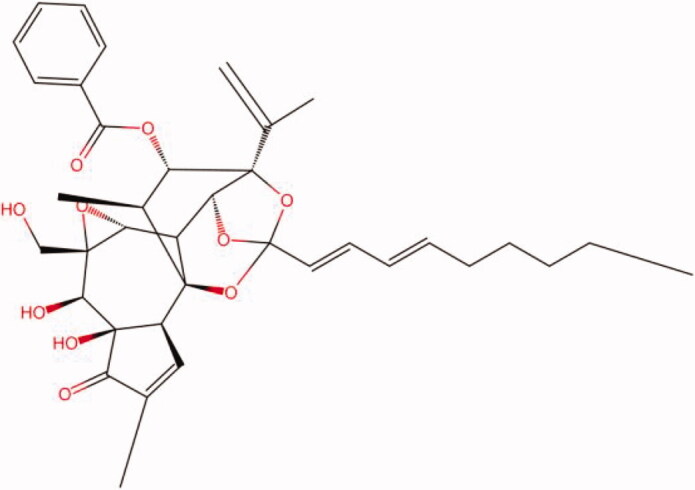

**Table 2.** Structural similarity between HC-067047 and ‘cold’ compounds from TCM (similarity > 0.5).

Molecule ID	Molecule name	Similarity	Structure
N/A	HC067047	1.00	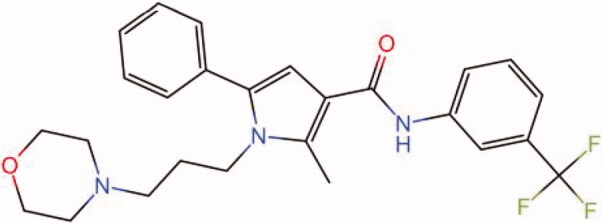
MOL002935	Baicalin	0.62	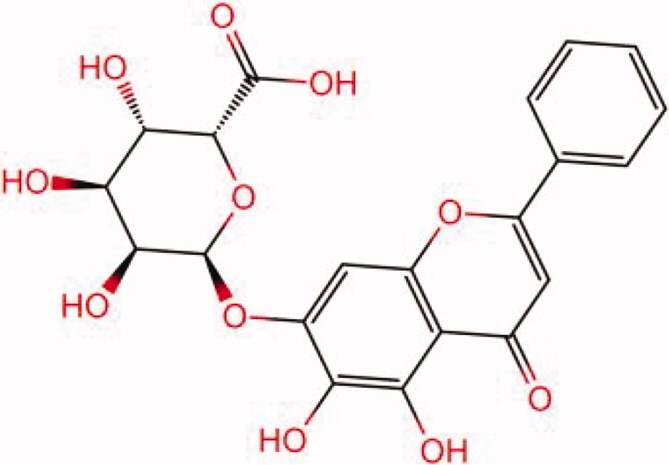
MOL005780	Blestrin	0.60	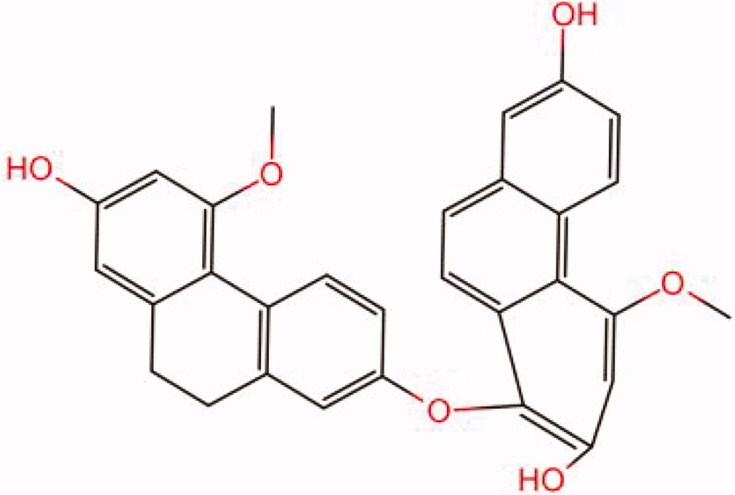
MOL001131	Phellamurin	0.58	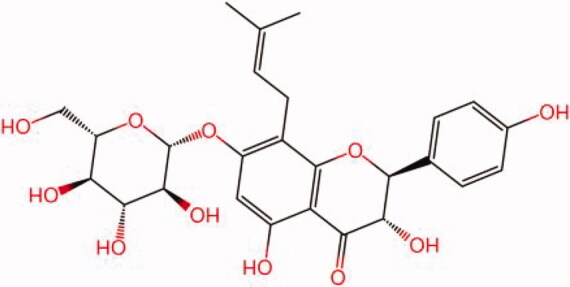
MOL005775	Blestriarene	0.58	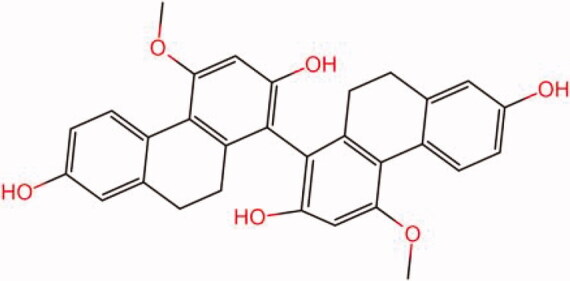
MOL002343	Tetrandrine	0.57	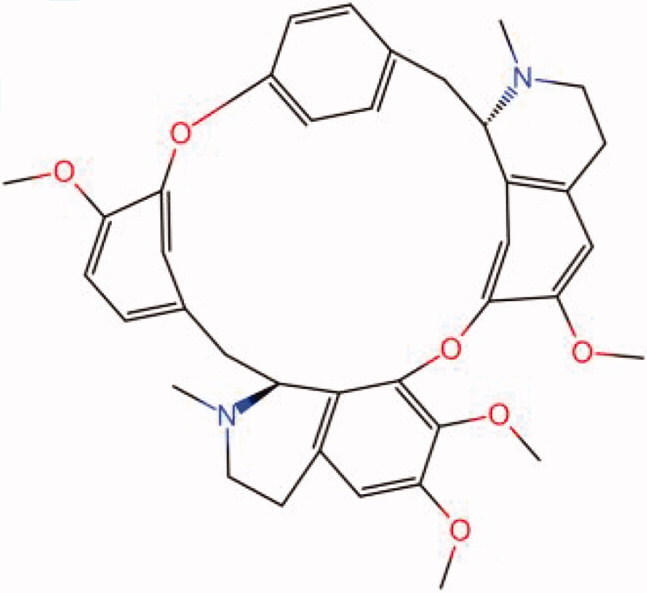
MOL002714	Baicalein	0.56	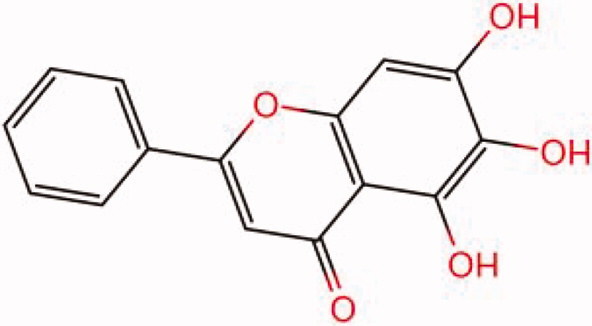
MOL008228	Andrographin	0.56	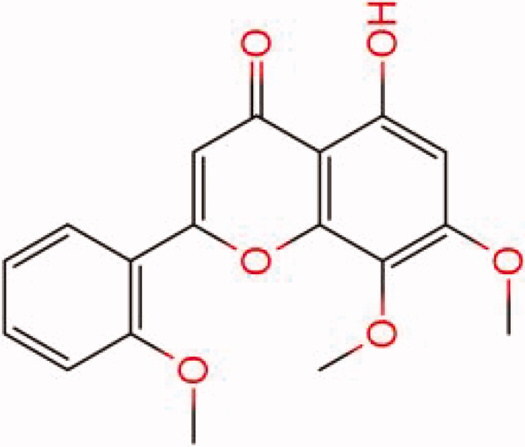
MOL006147	Alizarin-2-methylether	0.56	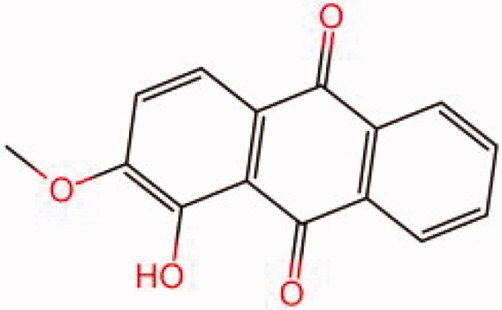
MOL007729	Shikonofuran	0.55	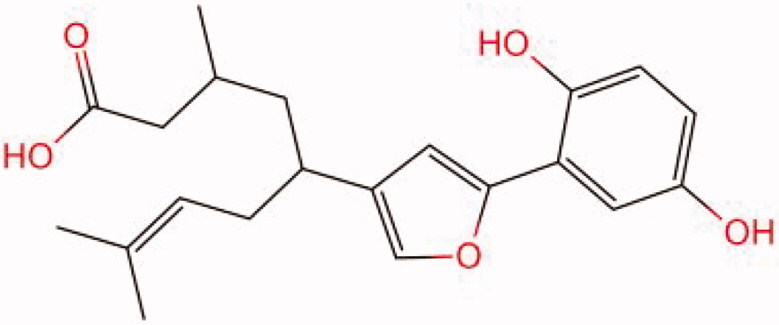
MOL002891	Magnoflorine	0.54	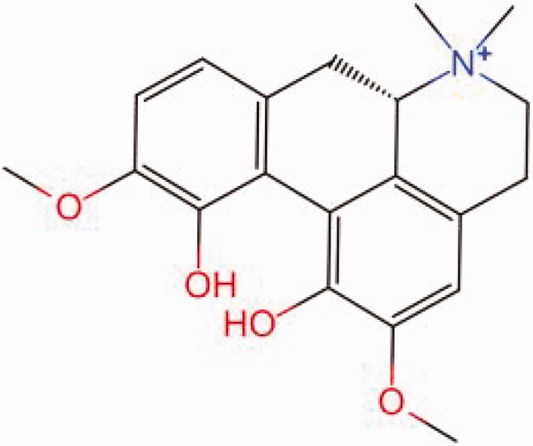
MOL002268	Rhein	0.52	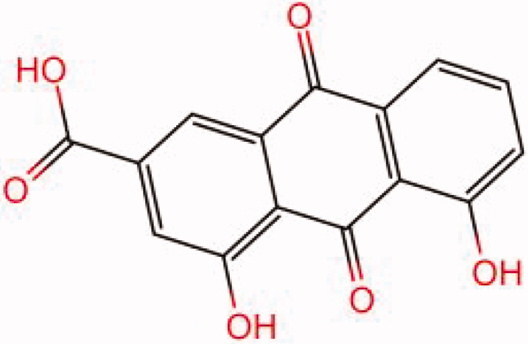
MOL000476	Physcion	0.52	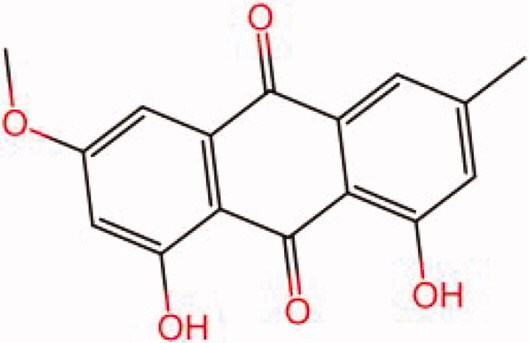
MOL001925	Paeoniflorin	0.51	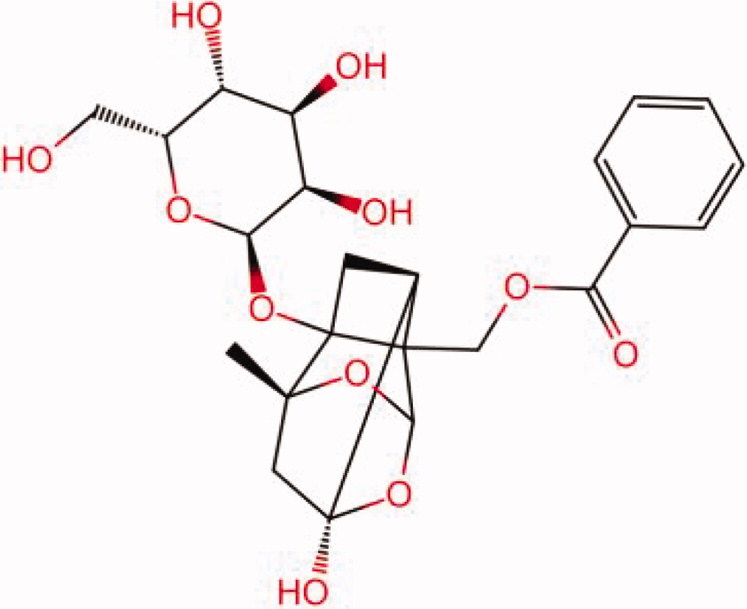
MOL001918	Paeoniflorgenone	0.51	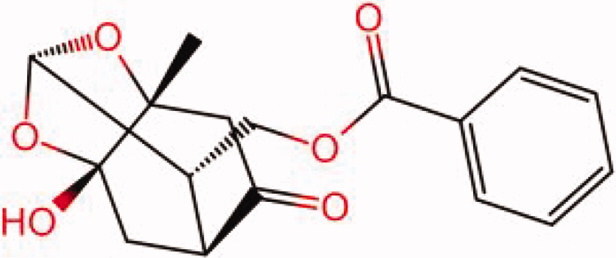

### SEM observation of A375 cells grown on thermal resistors

The detection of cellular temperature contributes to understanding the influence exerted by the external environment on cells, which not only can provide an understanding of various cellular events, but also may grasp the pathological state of the cells (Lowell and Spiegelman 2000; Braissant and Daniels 2011). The current cell temperature measurement is mainly conducted under room temperatures, vacuum, or make adherent cells suspensive (von Ah et al. 2009; Wang et al. 2011; Kucsko et al. 2013). Most of these detection methods are basically inconsistent with the normal growth conditions of cells, thereby affecting the status of cells and the reliability of the results.

Based on the deficiency of existing cell temperature measurement methods, our team developed a multi-channel, real-time, and *in situ* wireless temperature measurement system based on PT1000 thermal resistors. This system can achieve cell population temperature detection under the normal growth conditions of cells without damaging them during measurement. The wireless, real-time, high-throughput temperature detection technique allows the process of cells interacting with extracellular matrices to be monitored with a fast time resolution.

**Figure 2.**
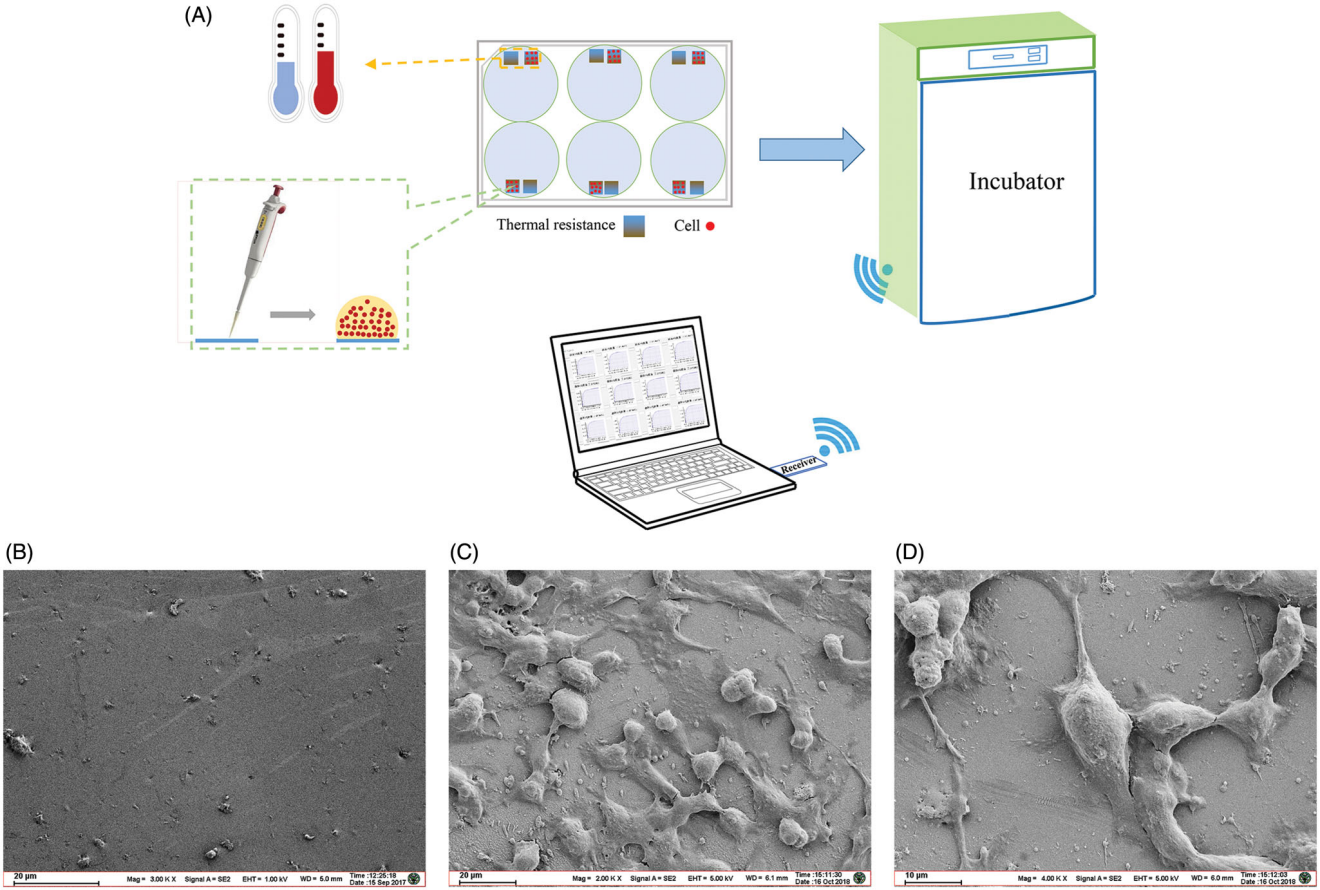
Schematic diagram of the temperature measurement experiment and observation of the thermal resistors surface with and without A375 cells growing by SEM. (A) Schematic diagram of the temperature measurement experiment. (B) Thermal resistors surface without cells. (C) Thermal resistors surface with A375 cells growing (100 ×). (D) Thermal resistors surface with A375 cells growing (200 ×).

Figure 2(A) shows the schematic diagram of the wireless temperature measurement system. Each hole of the six-well plate is composed of a reference PT1000 film resistor for culture medium temperature detection, and a PT1000 film resistor on which cells are seeded by a pipetting gun. Then the six-well plate is placed in an incubator for the purpose of cell cultivation. The interaction process between cells and extracellular matrices is monitored by the wireless, real-time, high-throughput temperature measurement system (Li et al. 2019a, 2019b). The growth state of A375 cells is shown in Figure 2(C,D), while Figure 2(B) presents the sensor surface without cells. From the figures, it can be seen that the cells seeded on the sensor are growing very well. The structure of A375 cells is stereoscopic, the shape is full, and the connections between cells are normal and tight (Figure 2(C,D)).

### The relationship between the thermogenesis of A375 and the opening/close state of TRPV4

**Figure 3.**
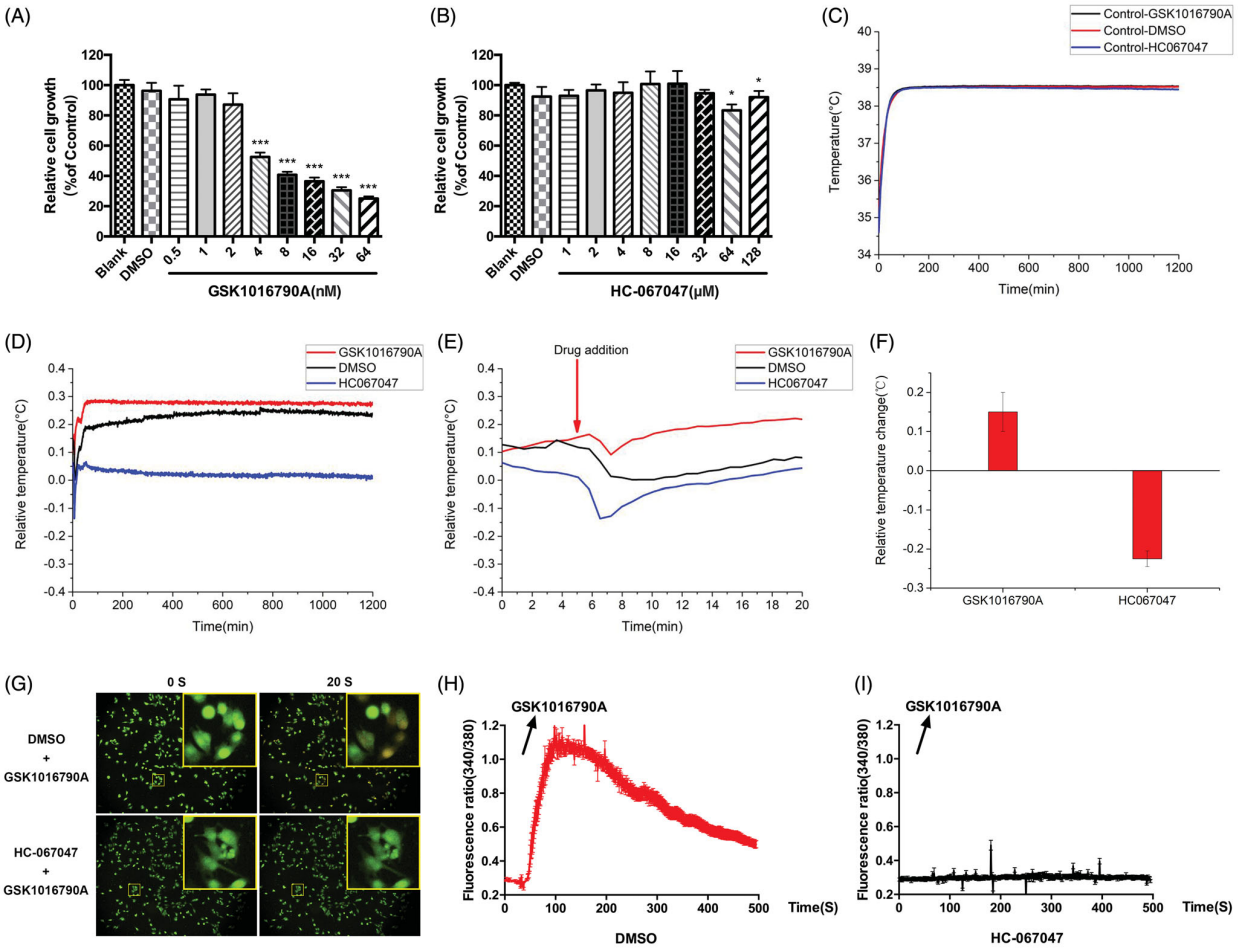
Effects of GSK1016790A and HC-067047 on A375 cell temperature and calcium influx. (A) Effect of GSK1016790A (0.5, 1, 2, 4, 8, 16, 32, and 64 nM) on the proliferation of A375 cells after 24 h intervention. (B) Effect of HC067047 (1, 2, 4, 8, 16, 32, 64, and 128 μM) on the proliferation of A375 cells after 24 h intervention. (C) Median temperature for GSK1016790A, DMSO of 2 μL, DMSO of 10 μL and HC067047 on A375 cells. (D) Temperature changes of A375 cells relative to median temperature for GSK1016790A and HC067047. (E) The local amplification of (D) which shows the dosing details. (F) Statistics of cell temperature changes for GSK1016790A and HC067047 compared to control conditions (*p* = 0.0002). (G) The calcium influx of A375 cells. The top and bottom is, respectively, the group pre-incubated with DMSO and HC-067047 30 min before stimulated by GSK1016790A; the left of 0 s is the calcium ion level in A375 cells without GSK1016790A, and the right 20 s is the calcium ion level after GSK1016790A was added. (H) Quantitative caption of intracellular calcium ion fluorescence intensity over time in A375 cells pre-incubated with DMSO 30 min before stimulated by GSK1016790A. (I) Quantitative caption of intracellular calcium ion fluorescence intensity over time in A375 cells pre-incubated with HC-067047 30 min before stimulated by GSK1016790A. Values are presented as mean ± SD (*p < 0.05, ***p < 0.005).

TRPs are closely related to energy metabolism, and metabolism is suggested to be related to cellular temperature changes. The opening/close state of TRPV4 could exert a significant effect on intracellular calcium. Therefore, it is the top priority to ascertain whether the opening/close state of TRPV4 channel will have impact on cell temperature. We conducted investigation into agonists and inhibitors of TRPV4. Firstly, cell proliferation and growth were assessed after GSK1016790A and HC067047 were treated, and the IC_50_ values are 8.363 nM and 816.4 μM, respectively. Then, we used concentrations exerting no effect on cell growth for subsequent experiments. The concentrations selected for GSK1016790A and HC067047 were 2 nM and 32 μM, respectively (Figure 3(A,B)). It was found that the cellular temperature was altered after disturbance to TRPV4. Our results indicated that the cellular temperature was totally different under the stimulation of GSK1016790A and HC-067047 (Figure 3(C–F)). We observed that GSK1016790A induced temperature rise in A375 cells, while HC067047 led to a cellular temperature decline. The insult of GSK1016790A to A375 cells led to an acute increase in cellular temperature (about 0.15 °C), which was maintained during the experimental period, while HC-067047 led to a decrease in cellular temperature (by about 0.2 °C), which was also sustained during the experimental period.

Since calcium ions are reported to be associated with the changes in cell temperature (Sun et al. 2017), and TRPV4 are reported as non-selective ion channel proteins which were mainly involved in a plethora of Ca^2+^-mediated cell functions, the cellular calcium influx evoked by the drug was examined in this study. From the results, it can be seen that GSK1016790A caused a calcium influx in A375 cells (Figure 3(G,H)), while this effect failed to be observed when HC-067047 was pre-incubated in advance (Figure 3(G, I)). The above experimental results indicate that the opening and closing of the TRPV4 channel can affect the temperature change of A375 cells, and this change may be caused by the calcium influx mediated by TRPV4.

### The cell temperature change caused by hypaconitine and baicalin acting on TRPV4

**Figure 4.**
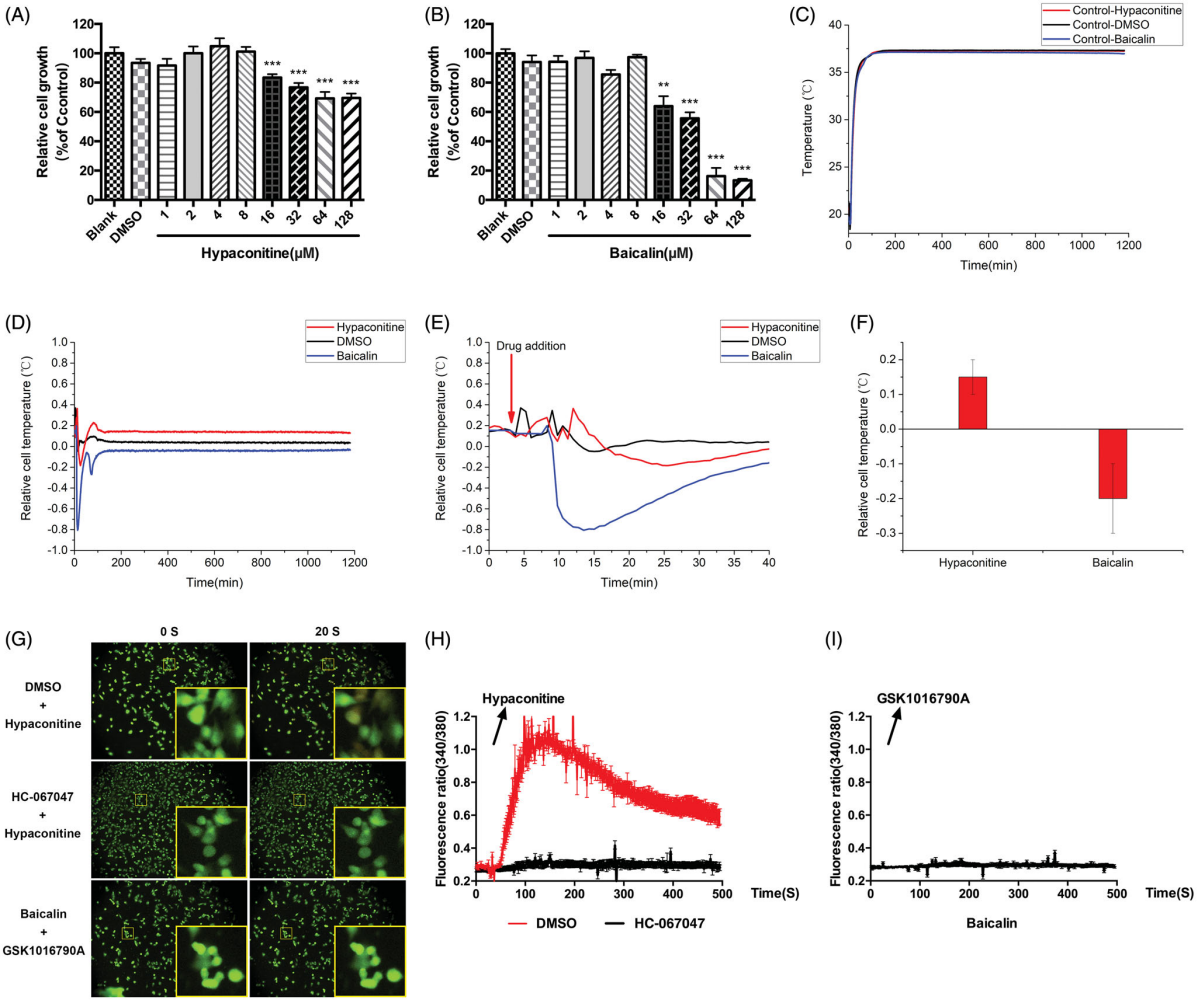
Effects of hypaconitine and baicalin on A375 cell temperature and calcium influx. (A) Effect of hypaconitine (1, 2, 4, 8, 16, 32, 64, and 128 μM) on the proliferation of A375 cells after 24 h intervention. (B) Effect of Baicalin (1, 2, 4, 8, 16, 32, 64, and 128 μM) on the proliferation of A375 cells after 24 h intervention. (C) Median temperature for hypaconitine, baicalin, and DMSO on A375 cells. (D) Temperature changes of A375 cells relative to median temperature for hypaconitine and baicalin. (E) The local amplification of (D) which shows the dosage details. (F) Statistics of cell temperature changes for hypaconitine and baicalin compared to control conditions (*p* = 0.0006). (G) The calcium influx of A375 cells. The top and middle is respectively the group pre-incubated with DMSO and HC067047 30 min before stimulated by hypaconitine; the bottom is the group pre-incubated with baicalin 30 min before stimulated by GSK1016709A; the left of 0 s was the calcium ion level in A375 cells without agonists, and the right 20 s was the calcium ion level after hypaconitine/GSK1016790A was added. (H) Quantitative caption of intracellular calcium ion fluorescence intensity over time in A375 cells pre-incubated with DMSO/HC067047 30 min before stimulated by hypaconitine. (I) Quantitative caption of intracellular calcium ion fluorescence intensity over time in A375 cells pre-incubated with baicalin 30 min before stimulated by GSK1016790A. Values are presented as mean ± SD (**p < 0.01, ***p < 0.005).

The same as above, cell proliferation and growth were assessed and the IC_50_ values of hypaconitine and baicalin for A375 cells are 286.4 μM and 29.84 μM, respectively. Then, we selected the concentration that had no effect on cell growth for subsequent experiments. The concentration of hypaconitine and baicalin was set to 8 μM (Figure 4(A,B)). Subsequently, the effects of hypaconitine and baicalin on cell temperature were further observed. Surprisingly, hypaconitine and baicalin exerted a similar effect to GSK1016790A and HC067047, respectively. These proof-of-concept cell analysis study results demonstrate that the cellular temperature of A375 was increased under the stimulation of hypaconitine while baicalin led to a decrease in cellular temperature (Figure 4(C–F)). The drug of hypaconitine insult led to an acute increase in cellular temperature (about 0.15 °C), which was maintained during the experimental period, while baicalin caused a decrease in cellular temperature (by about 0.2 °C), which was also maintained during the experimental period.

As the effects of hypaconitine and baicalin on cell temperature are consistent with the agonist-GSK1016790A and the inhibitor-HC067047 of TRPV4, do they also exert effect through TRPV4 channel? The results of Ca^2+^ imaging showed that hypoaconine caused calcium influx in A375 cells (Figure 4(G,H)), and this effect was abrogated by HC067047. It was also demonstrated that hypoaconine led to the opening of TRPV4 of A375 cells, just as GSK1016790A did. GSK1016790A failed to cause calcium influx when cells were pre-incubated with baicalin, indicating that baicalin can inhibit the opening of TRPV4 channel (Figure 4(G,I)), just as HC067047 did. Collectively, the ‘hot’ compound hypaconitine may change the cell temperature by activating TRPV4 to increase intracellular calcium, while the ‘cold’ compound baicalin probably decreased the cell temperature by suppressing the opening of TRPV4.

## Discussion

Our research investigated the potential ‘cold/hot’ property of compounds in TCM from the perspective of cellular and molecular biology for the first time. The ‘cold/hot’ properties of TCM are important basis for syndrome differentiation and treatment. However, the alleged ‘cold/hot’ properties of TCM is a highly metaphysical theory and it is difficult to identify a scientific way to prove and characterize it. Besides, there have been many related studies on the ‘cold/hot’ properties of TCM, but the inconsistency of research methods has resulted in a significant difference in the results (Liu et al. 2008; Wang et al. 2016; Fu et al. 2017; Jia et al. 2017; Huang et al. 2018). To date, it remains unlikely to obtain the common internal law of the “cold/hot” herbs. As indicated by our study, the ‘cold/hot’ property of TCM may be associated with the changes to heat production in cells caused by temperature sensitive receptors-TRPs.

According to the established TCM theory, ‘cold/hot’ property of TCM was emphasized through the relationship between environment temperature and the actual feeling. In a cold environment, our body feels cold and tends to move to a relatively warm environment in order for survival. The perception and assessment of temperature changes inside and outside the body is primarily achieved by temperature sensitive receptors, which are the family of TRP receptors (Feng 2014; Millqvist 2015). Besides, the existing studies on ‘cold/hot’ properties of TCM are mainly focussed on the nervous system, endocrine system and energy metabolism. The influence exerted by ‘cold/hot’ herbal drugs on the energy metabolism is achieved by affecting the activity of Na^+^–K^+^-ATPase (Zhao et al. 2016). Increased intracellular calcium concentration can promote cell energy metabolism (Glancy and Balaban 2012). As ion channels, TRPs participate in a plethora of Ca^2+^-mediated cell functions. As reported in the relevant studies, the intracellular Ca^2+^ concentration is relevant to the intracellular thermogenesis (Uchida et al. 2018). The cellular temperature change is closely related to life activities and pathological status of human body (Braissant and Daniels 2011). The thermogenesis capacity of cells is supported by maintaining active metabolism and sacrificing fuel efficiency (Zhang and Bi 2015; Chouchani et al. 2019). Some of these are related to ATP hydrolysis and maintenance of the cationic gradient across membranes (Lowell and Spiegelman 2000). Under compounds or some other stimulations, cells have a potential to exhibit acute intracellular temperature alterations due to the changes in metabolic levels (Zhang et al. 2016). Whether the opening of the TRPs channels could cause cellular temperature change still await to be explored, and whether the thermogenesis effect of the ‘cold/hot’ herbal drugs on cells is regulated by TRPs remains unclear. On account of the above background, our team developed a new system for measuring temperature changes at the cellular level. As reported in our previous study (Li et al. 2019a, 2019b), the platinum sensor and multi-channel acquisition system can be used to determine the temperature changes in cells in their original state. The wireless, real-time, high-throughput temperature detection method is particularly suitable to evaluate the thermogenic ability of cells without disruption caused to their interaction with other matter or organisms.

As an important member of temperature sensitive receptors, TRPV4 has a certain level of basal activation at room temperatures and is widely distributed in various organs. We then screened cells with a high expression level of TRPV4 through CCLE analysis and experimental verification. The CCLE database includes genetic information on more than 1000 cell lines and has been visualized, including copy number, mRNA expression (Affy, RNAseq), and so on (Barretina et al. 2012, 2019). It allows us to perform a predictive analysis of the gene expression of cells in silicon. The analysis manifested that melanoma cell lines showed a high expression level of TRPV4, of which A375 was the highest in experimental verification. To verify the relationship between TRPV4 and cell heat production, we used the agonist and antagonist of TRPV4 to conduct a preliminary study. GSK1016790A, as a specific agonist of TRPV4, can induce calcium influx. Eventually, the temperature of A375 cells was raised and maintained at a certain level. By contrast, when the TRPV4 was inhibited by HC067047, the temperature of the cells showed a downward trend and remained unchanged during the experiment. Mammals could sense hot and cold through TRP channels, despite that the mechanism remains unclear. Combined with our experimental results, it is speculated that TRPV4 may cause changes in intracellular calcium concentration through the opening/closing of its channels, thus eventually alter the heat production in cells. Among which, energy metabolism perhaps plays an important role in the heat production of cells.

**Figure 5.**
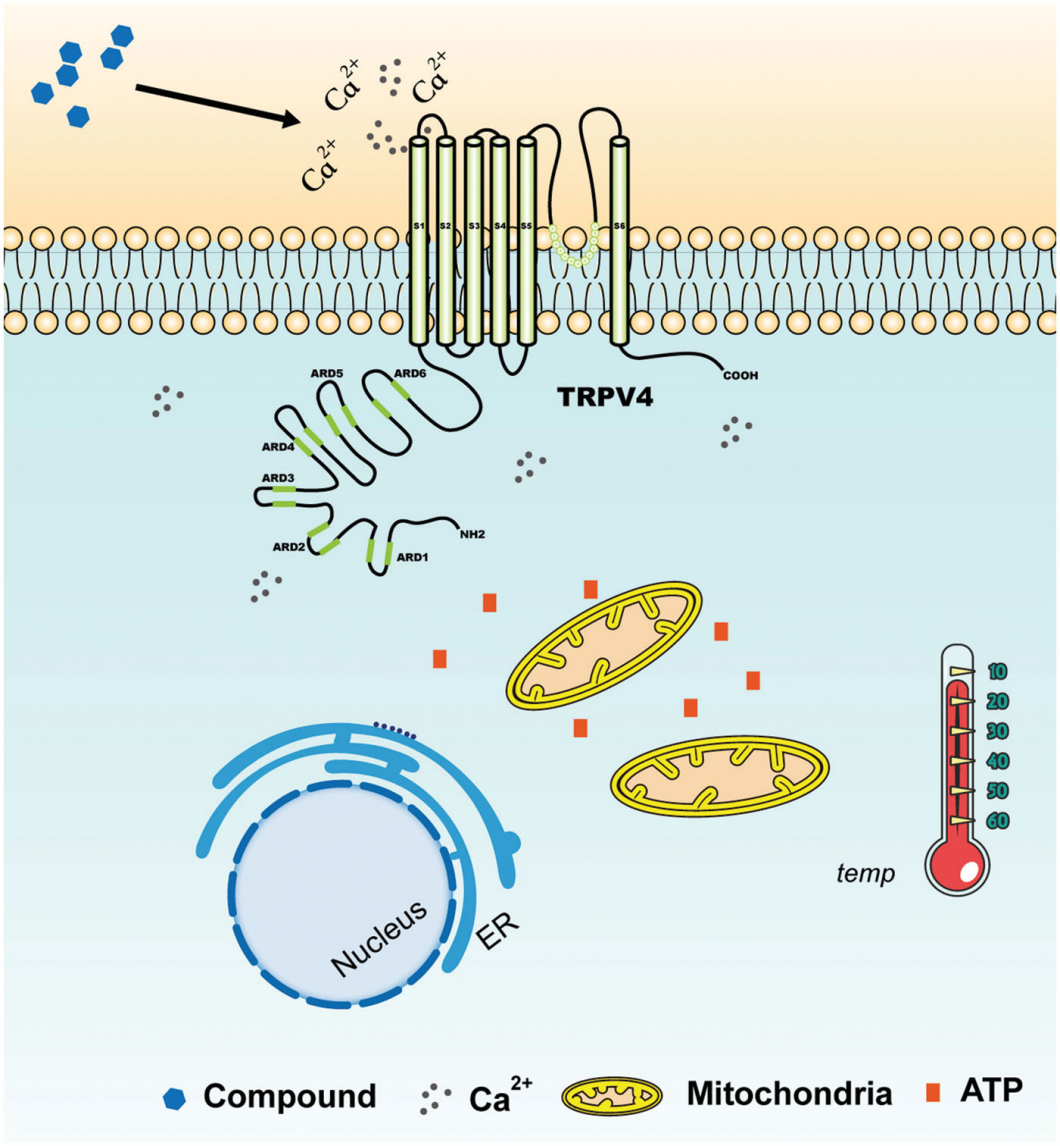
Sketch of temperature change caused by TRPV4. The local inset refers to the locally enlarged display of the graphs. The compounds acting on TRPV4 which has the capability to open the channel can cause extracellular calcium ions to flow in, and the intracellular calcium ion concentration will be significantly increased. An increase in the intracellular calcium ion concentration enhances the energy metabolism level of the cells by affecting mitochondria and some other organelles, thereby eventually raising the cell temperature. However, the opposite effect will occur if the TRPV4 channel is shut down.

As a representative ‘hot’ compound screened from TCM, hypoaconine can produce acute calcium influx and raise cell temperature, which is similar to GSK1016790A, while the representative ‘cold’ compound baicalin can restrain calcium influx mediated by TRPV4 and cause a down-regulation of cellular temperature just as HC067047. The effect of hypaconitine and baicalin on cell temperature prompted that the ‘cold/hot’ properties of compounds may be classified by cellular temperature detection. Therefore, we infer that compounds acting on TRPs may have the capability to open the channel, thereby causing extracellular calcium ions to flow into the cells and significantly increasing intracellular ion concentration. An increase in the intracellular calcium ion concentration can enhance the cellular energy metabolism level by affecting mitochondria and some other organelles, thereby eventually raising the cell temperature (Glancy and Balaban 2012). Conversely, the opposite effect will occur if the channel is closed (Figure 5). Consequently, we presumed that the ‘cold/hot’ properties of TCM may be related to TRP-mediated cell heat production.

Certainly, due to the limitations of our research, the current experimental results failed to fully explain the ‘cold/hot’ properties of TCM. These results have not been validated in normal cells, and fewer types of cells and compounds have been explored. It requires more exploration and discussion to be conducted in the future experiments. Our research only hopes to provide a new mentality and method for revealing the ‘cold/hot’ properties of TCM. It is expected that we can clarify the objectivity of the difference between the “cold” and “hot” herbal drugs, and establish a more recognized evaluation method to distinguish them.
